# A novel subspace outlier detection method by entropy-based clustering algorithm

**DOI:** 10.1038/s41598-023-42261-4

**Published:** 2023-09-15

**Authors:** Zheng Zuo, Ziqiang Li, Pengsen Cheng, Jian Zhao

**Affiliations:** 1 Chengdu University of Information Technology, Chengdu, China; 2 The National Computer Network Emergency Response Technical Team/Coordination Center of China, Beijing, China; 3 School of Cyber Science and Engineering, Sichuan University, Chengdu, China

**Keywords:** Engineering, Mathematics and computing

## Abstract

Subspace outlier detection has emerged as a practical approach for outlier detection. Classical full space outlier detection methods become ineffective in high dimensional data due to the “curse of dimensionality”. Subspace outlier detection methods have great potential to overcome the problem. However, the challenge becomes how to determine which subspaces to be used for outlier detection among a huge number of all subspaces. In this paper, firstly, we propose an intuitive definition of outliers in subspaces. We study the desirable properties of subspaces for outlier detection and investigate the metrics for those properties. Then, a novel subspace outlier detection algorithm with a statistical foundation is proposed. Our method selectively leverages a limited set of the most interesting subspaces for outlier detection. Through experimental validation, we demonstrate that identifying outliers within this reduced set of highly interesting subspaces yields significantly higher accuracy compared to analyzing the entire feature space. We show by experiments that the proposed method outperforms competing subspace outlier detection approaches on real world data sets.

## Introduction

Outlier detection techniques have been widely used in a variety of practical application domains, such as fraud detection for credit cards^1^, cyber-attack detection^2^, and cancer diagnoses^3^. It is critical to improve outlier detection in order to develop better strategies in those domains. Definitions of outliers vary in different perspectives. A fundamental definition of an outlier is that “an observation which deviates so much from other observations as to arouse suspicions that it was generated by a different mechanism”^4^. Another way of defining outlier is “An outlier is an observation (or subset of observations) which appears to be inconsistent with the remainder of that dataset”^5^. These definitions motivate the computer science community to design new techniques to detect outliers. Among which two typical methods for are distance-based and density-based methods^6–8^. The basic assumption of distance-based approaches is that outliers are far away from their neighboring objects while the basic assumption of density-based methods is that the densities of outliers are very low.

**Figure 1:**
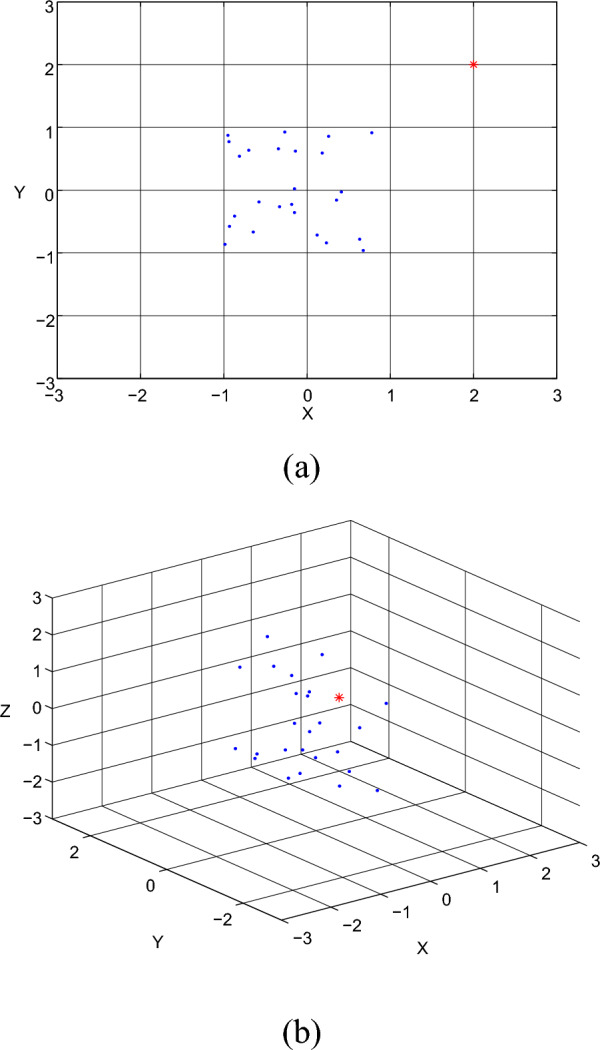
(**a**) An outlier is clear in a subspace (**b**) The outlier is more difficult to be detected in the full space.

Existing full space outlier detection methods do not work well with high dimensional data. Both distance-based and density-based full-space approaches work well with low-dimensional data. However, as dimension increases, these approaches are likely to lose effectiveness due to distance concentration^9^. That is, data points become similar as dimensions increase. This phenomenon is also known as “curse of dimensionality^10^”. This makes detecting outliers ineffective when the detection is based on either distance or density in the high dimensional data space. Further, outliers may only exist in some subspaces but not in the full space. The detection of these outliers in the full space is impossible since many irrelevant dimensions are involved. Figure 1 shows an example where an outlier may not be detected in the full space, but is easily discovered in a subspace. This example explains why many classical outlier detection methods work well on relatively low-dimensional data sets, but fail with high dimensional data.

Subspace outlier detection has become a new promising approach for finding outliers in high dimensional data sets. Subspace outlier detection methods project data points into lower dimensional spaces where the discovery of outliers is performed. The projection of data points into lower spaces alleviates the problem of “curse of dimensionality”. Furthermore, subspace outlier detection is able to detect outliers which are undetectable in the full space due to irrelevant attributes interference. However, subspace outlier detection introduces its own problems. Firstly, the sheer number of subspaces make exhaustive search impossible. Finding outliers in all subspaces using an exhaustive searching algorithm is a NP problem^11,12^. Even the problem of dimension selection, i.e. finding the appropriate sets of dimensions to form up a subspace, is a NP-hard problem^13^. Secondly, a large number of repetitive tests on whether a data point is an outlier in subspaces may produce many false discoveries (type I errors). In other words, this test process decreases the precision of subspace outlier detection. In summary, the discovery of right subspaces for outlier detection is still an open challenge^14^.

It is a general consensus that a dense subspace which density distribution deviates significantly from an expected density distribution is good for outlier detection.

Distances to data points in dense regions are used in a benchmark work in subspace outlier detection. SOD (Subspace Outlier Degree)^15^ measures the outlierness of a data point using the distance in a subspace where the closest neighbors exhibit a low variance. In other words, in a preferred subspace, the closest neighbors are in a dense region, and this indicates that they are likely generated from the same mechanism. A data point deviating greatly from its closest neighbors is likely to be generated from another mechanism and hence is considered as an outlier.

Density distribution is used to select right subspaces for outlier detection in following up work, statistically selection of relevant subspace projects^16^ and high contrast subspaces^17^. A key concept of both works is the density contrast between outliers and the residual data points. Such a contrast is maximized when the residual data points are in dense regions, and the density distribution is significantly deviated from the expected density distribution of uniformly distributed subspace.

Some other measures, such as OutRank^18^ and OutRes^19^, directly make use of the information of clusters in subspaces, and the clusters are formed by dense data points in subspaces already.

Density distribution is a crucial criterion for assessing whether data points in a subspace can form clusters or not. An early work of subspace clustering^20^ has studied the criteria for searching for subspaces that exhibit good clustering properties. The first criterion is high coverage which requires that the most data points are in clusters and this leaves a small number of possible outliers. The second criterion is high density that enforces the quality of clusters in subspaces and an entropy-based criterion ensures that the density distribution deviates from the expected distribution of uniformly distributed subspace. The above two criteria are consistent with the measures for outlier detection in subspaces discussed above. The third criterion is correlation of dimensions and this criterion is used to remove redundant subspaces. Some high dimensional subspaces do not improve the quality of clusters over its lower dimensional subspaces and hence they should not be considered. The authors have derived a set of coherent entropy-based criteria for searching and selecting the subspaces satisfying the three criteria.

We hypothesize that entropy-based criteria developed in^20^ are good for finding quality subspaces for outlier detection and the simple and coherent entropy-based criteria support an efficient algorithm. In this work, we propose an intuitive definition for measuring and comparing outlierness of data points in subspaces, and link outlier detection in subspaces with the entropy-based criteria for searching for clustering subspaces. Integrating the subspaces produced by the entropy-based criteria and our outlier ranking measure, we propose an efficient subspace outlier detection method EPOD (*E*ntropy-based subsPace *O*utlier *D*etection). EPOD is more accurate than other benchmark outlier detection methods (or at least as accurate), and is faster than other subspace outlier detection methods. The parameters of EPOD are easy to set and we demonstrate that the performance of EPOD is stable over a wide range of parameter settings.

## Problem definition

In this section, firstly we formalize the problem, then investigate the interaction between subspace clustering and criterion of outlier detection. Afterwards, we explicate the main motivation of this paper, and then we come up with our intuitive method.

The purpose of outlier detection is to discover records which deviate from the majority of records in a data set. Each record is constituted by a number of attributes depicting features and numerical information of this record. We preliminarily focus on unsupervised method.

Thus, our goal is to construct an approach to detect outliers with some interest attributes in datasets instead of all attributes.

Given a data set *D* = {*x*_1_*,x*_2_*,…,x*_*n*_} in dimension *A* = {*A*_1_*,A*_2_*,…,A*_*m*_}. The following definitions are based on the full space *A* as well as a subspace *A*^′^ ⊆ *A*.

### Definition 1 (k-distance)

*The k-distance of data point x*_*i*_* is the farthest distance among the k-nearest neighbors*^21^* of data point x*_*i*_*, denoted as d*^*k*^(*x*_*i*_)*. When the distances of x*_*i*_* to all other data points in D are sorted by the distance ascending order, d*^*k*^(*x*_*i*_) *is the k-th value.*

The *k*-distance indicates the closeness of a data point to its neighbors. In general, if a data point is very close to its neighbors, the *k*-distance will be small, and vice versa. If a data point is extremely far away from its neighbors, we have a reason to suspect it as an outlier.

### Definition 2 (z^k^ value)

*Let *$\sigma_{o} = \sqrt {\frac{{\sum\nolimits_{i = 1}^{n} {\left( {d^{k} \left( {x_{i} } \right) - \overline{d^{k}} \left( {x_{i} } \right)} \right)^{2} } }}{n - 1}} .$* z*^*k*^* value of x*_*i*_* is defined as* $z^{k} \,\left( {x_{i} } \right) = \frac{{d^{k} \left( {x_{i} } \right) - \overline{d^{k}} \left( {x_{i} } \right)}}{{\sigma_{o} }}.$

Note that we use *σ*_*o*_to differentiate it from *σ* since *σ*_*o*_is relative to the origin whereas *σ* is relative to the mean of *k*-distances in a subspace. We consider that the origin is an expected center of *k*-distances. We define *z*-values relative to the ideal center instead of the real mean location. Such process makes the *z*-values less affected by the mean locations which vary a lot in different subspaces and hence *z*-values are more comparable from different subspaces.

Our objective is to find top *N* data points with the largest *z*^*k*^-values in all subspaces. These data points are distant from their closest neighbors in a data space, and hence we consider them as outliers.

To improve the chance of detecting data in this area, equally detecting points with a large *z*^*k*^value, we need to find a subspace with a small *σ*_*o*_. Firstly, given the same *k*-th distance, a small *σ*_*o*_will make the *z*-value large. Secondly, given a fixed *z* value, a small *σ*_*o*_will make the probability of the data point occurring large. Assume that we can group *l* dense data points in a subspace into a slice of clusters. Remainder (*n* − *l*) spare data points do not belong to any cluster. We can rewrite the *σ*_*o*_as Eq. (1).1

The first part stands for data points in the clusters, and the second part stands for data points outside clusters. *d*^*k*^(*x*_*i*_) in the second part is larger than *d*^*k*^(*x*_*i*_) in the first part since data are sparse outside clusters. To obtain a small *σ*_*o*_, we have the following two options.

1. Reduce distance *d*^*k*^(*x*_*i*_) in the first part. This requirement is equivalent to the high density of subspace clusters;
2. Minimize the number of data points in the second part. This requirement is equivalent to the high coverage of subspace clusters.

Therefore, the subspaces that we should look for outliers are those containing clusters of high density and high coverage. Cheng, Fu and Zhang^20^ studied the relationship between subspace entropy and cluster coverage and density, and concluded that subspace entropy is a good indicator of cluster density and coverage,which is shown in Eq. (2).

2 $$H\left( {A_{1} , \ldots ,A_{p} } \right) = - \sum\nolimits_{{a_{1} \in A_{1} }} { \cdots \sum\nolimits_{{a_{1} \in A_{p} }} {p\left( {a_{1} , \ldots ,a_{p} } \right)} } \log \,p\left( {a_{1} , \ldots ,a_{p} } \right),$$

where *p* ≤ *m* (the dimension of a data set) means a subspace. Data space is partitioned to form a grid and the probability is estimated by the density in each cell. A low entropy generally indicates high density and high coverage of clusters in the subspace. Interesting subspaces are those with low entropy. In work^20^, another criterion, correlation of dimension, is also discussed. The correlation has not been mentioned in our previous discussions, but it is necessary for finding interpretable outliers. Note that the correlation in this paper does not mean statistical correlation, being correlated, but not fully independent. There are a huge number of subspaces for a median dimensional data set. The huge number will result in a noticeable number of interesting subspaces by chance, where data objects happen to project to a dense and high coverage space. These clusters are not interested by users, and neither outliers from the subspace.

The correlation of attributes can be measured by entropy. An interesting criterion of a subspace is presented in^20^ as Eq. (3).

3 $$interest\,\left( {\left\{ {A_{1} , \ldots ,A_{p} } \right\}} \right) = \sum\limits_{i = 1}^{n} {H\left( {A_{i} } \right) - H\left( {A_{1} , \ldots ,A_{n} } \right)} .$$

The higher the interest, the stronger the correlation. The above interest criterion prefers low dimension subspaces. In many cases, we need higher dimension subspaces. A revised criterion called *interest gain* for measuring the increase in correlation is introduced in^20^, as Eq. (4).

4 $$interest \, gain\left( {\{ A_{{1}} ,...,A_{p} \} } \right) \, = interest\left( {\{ A_{{1}} ,...,A_{p} \} } \right) \, - max_{i} \left\{ {interest\left( {\left\{ {A_{{1}} ,...,A_{p} } \right\} \, - \, \{ A_{i} \} } \right)} \right\},$$

The interest gain is used as a criterion for finding subspaces that have strong correlation among the attributes forming the subspaces. By jointly considering the density and coverage of clusters in a subspace and the correlation of attributes forming the subspace. We have the following searching heuristic.

Heuristic 1 *Searching for subspaces with low entropy and high interest gain.*

For easy description, we call those subspaces with low entropy and high interest gain as interesting subspaces. Theoretically, we should search for outliers with high *z*^*k*^-values in subspaces. However, we are unable to search for all subspaces. We need to consider meaningful subspaces with dense and high coverage clusters, with strong correlation among attributes. We called these subspaces as interesting subspaces.

As in Condorcet’s Jury theorem: One or another judgment about an observation might be wrong, but the majority might still be right, as long as the judgments are, overall, somewhat reliable and every number decides independently from the others. So, to alleviate the Type I error problem, we introduced top-n style outlier detection methods.

### Definition 3 (Top ***N*** Outliers)

*Given an object o* ⊆ *D, Let Tbe a subset of D. A*^′^_*i*_*is a subspace of D, i* ≤ 2^*m*^ − 1*. The rank value r*_*k*_(*o,D*) *is the sum of the z-values in A*_*i*_*. T with the size of N in D, if there not exist objects x* ∈ *T and y* ∈ *D such that r*_*k*_(*y,D*) > *r*_*k*_(*x,D*)*, Then T is said to be the set of the top N outliers in D.*

The search for top *N* outliers becomes the search for top *N* outliers in the most interesting subspaces. Note that, top *N* outliers is equivalent to top *N*% of a data set. This process not only makes the searching possible, but also makes the detected outliers meaningful and interpretable to varying quantity of data. Moreover, we reduce the chance of false discoveries from multiple tests in a huge number of subspaces.

## Subspace outlier detectionmethod by entropy-based clustering algorithm

In this section, we discuss our proposed algorithm to discover top *N* outliers in most interest subspaces. Based on the intuition behind the candidate generation procedure in this algorithm, we also investigate the criterion of ranking interesting subspaces. We use interest gain to calculate the entropy property of the clustering in subspaces, and use distance-based outlier score, to identify the candidates of the top-n outlier. With the concepts described in the previous subsections, we can develop the major steps of the proposed method are outlined as follows:

1. Search potential subspaces with good clustering;
2. Rank most interesting subspaces by analyzing the entropy characterizations that may have higher percentage of outliers;
3. Rank the top *N* outliers by using distance-based or density-based algorithms in most interesting subspaces;

### Search potential subspaces with good clustering

A bottom-up method is used to find the interesting subspace by using Apriori-like method, which is similar to the method in^20^. The procedure of searching method is as follows. The algorithm first finds one-dimensional subspaces whose entropies *h*(*.*) are less than the threshold value *ω*. Then, based on these subspaces, we construct the candidate (*k* + 1)-dimensional subspaces and check them whether the entropy is less than the threshold value *ω*. Keeping these subspaces with interest gain greater than *ϵ*, and repeat the process again until the more subspace can be found whose entropy is less than *ω*. We refer to all of these subspaces as candidate interesting subspaces.

### Ranking for most interesting subspaces

Entropy criterion is especially good for clustering due when lacking of intuitive definition of distance in high dimension for record values. Hence, we utilize entropy property of subspace as the main criterion for sorting the subspaces which we discussed in previous section. By sorting the entropy values of interesting subspaces, we are able to obtain a list of subspaces with the most possible outliers embedding in. Additionally, we set up a dimensional pruning rule in our algorithm. That is, when the dimension of candidate *C* is greater than 5, the iteration of the algorithm stops. By sorting the entropy values of interesting subspaces, we are able to gain a list of subspaces with the most possible outliers embedding in.

### Majority voting for top N outliers n

In this algorithm is typically far smaller than the cardinality of dataset. Since outliers normally take up very small proportion in whole records. Instead of a binary outlier indicator, top-n outlier methods provide a ranked list of objects to represent the degree of outlierness for each object. The users (domain experts) can re-examine the selected top-n outlier to locate real outliers. Since this detection procedure can provide a good interaction between data mining experts and users, top-n outlier detection methods become popular in real-world applications. Distance-based, top-n Kth-Nearest Neighbor distance^21^ is a typical top-n style outlier detection approach. In order to distinguish from the original distance-based outlier detection method in^22^, we denote *K*th-Nearest Neighbor distance outlier as top-n *k*NN in this paper. In top-n *k*NN outlier, the distance from an object to its *k*-distance indicates outlierness of the object. Intuitively, the larger the *k*-distance is, the higher outlierness the object has. Top-n *k*NN outlier regards the n objects with the highest values of *k*-distance as outliers. We integrated outlierness scores of points from different subspace by summing up standardized Top-n *k*NN.

### Complexity of algorithm

In step 1, we adopt Enclus^20^ as a baseline for our algorithm. Since the number of interesting subspaces is normally not very large, the selection of the most interesting subspace does not take much time. Apart from these costs, the time complexity of the algorithm is determined by ENCLUS_SIG procedure. In addition, ENCLUS SIG has been shown reasonably scalable to the size of the dimension^20^. Step 2–3, pruning interesting subspace could be done in linear complexity which is *O*(*pn*). In step 4, querying the k-nearest neighbors, takes the majority of the computational load. Naively, the runtime of this step is *O*(*n*^2^). Fortunately, the implementation in ELKI is optimized by R*-Tree index, which can perform nearly at *O*(*n*log*n*). Overall, our proposed algorithm is not heavily affected by thresholds *ω* and *ϵ* (for interest and interest gain) significantly since these two parameters can be set in a proper range of values to balance effectiveness and efficiency, and this will be discussed in IV-D. And the pseudo code algorithm is listed in Algorithm1. In addition, the time complexity for finding top *N* outliers in the most interesting subspaces is *O*(*pn*log*n*), where *p* is the number of most interesting subspace and *n* is the number of all data points in *DB*. Data points in every most interesting subspace are sorted to find the top *N* outliers.

The parameter *k* is used to compute *k*NN distance. The algorithm is sensitive to the choice of *k* as all *k*-NN based methods. To demonstrate the impact on choosing *k*, we performed a few experiments on each real-world data set in order to find out the best *k* value for full space *k*-NN outlier detection method. Results are listed in Section VIII. In addition, A guideline for determining a good value for k is discussed in semi-supervised clustering field^23^ which is beyond this article.

*P*, which indicates the proportion of subspaces, is used to define an appropriate amount of interesting subspaces; *ω*(entropy thresholds) indicates subspace with dense clustering and good coverage; *ϵ*(interest and interest gain) is used as a threshold to prune subspace with attributes giving certain correlation; In subspace selection process, at each iteration, the interesting subspaces and candidate subspaces are included in the list *I*. Since the subspace in candidate set are regarded as being deviant from the objects in the interesting list, they may be regarded as interesting at the corresponding iteration. After one iteration, by generating next dimensional, any candidate subspace could find an additional attribute to form up a new interesting subspace.

The above constitute the primary three steps of our proposed method along with analyzes of the algorithm’s complexity. Subsequently, the pseudocode for the EPOD algorithm isshown in Table 1.

**Table 1:**
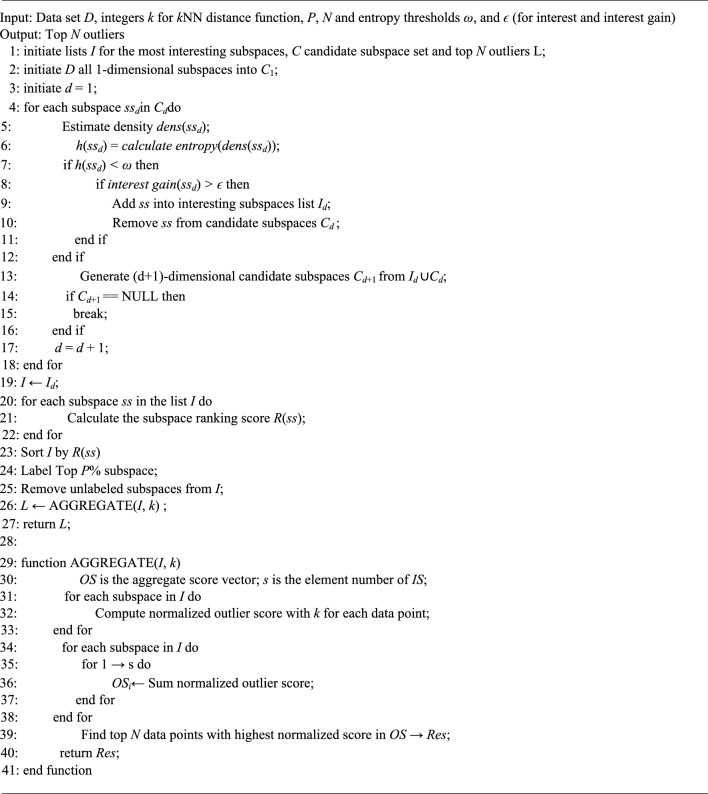
EPOD: Entropy based mining top *N* outliers in the most interesting subspaces.

## Experimental results

To evaluate the effectiveness and scalability of our proposed method, we prepare 8 real-world data sets and a group of synthetic data sets. Comparisons between the proposed method and other state-of-the-art subspace method and full-space methods. All experiments were performed on Intel® i7 4770 K quad-core CPU and clocked at 4.5 GHz with 16G of memory running on Ubuntu (64bit 14.04 LTS). Each Java JVM memory allocated up to 8 GB. Algorithms utilized in experiments are programmed in Java language. Additionally, due to some algorithms only proposed their methods but lack of some detailed implementation we employed implementations from the famous outlier detection framework ELKI^24^. To evaluate performance of scalability, we implemented our algorithm in Java and performed on the same workstation. Although we can partially parallelize our algorithm, for the sake of fairness, we deploy our method as a plug-in with single working thread in ELKI when do comparison with other algorithms.

In following experiments, we will show that we are able to discover outliers in a small number of most interesting subspaces than in the full space and that the proposed method is promising to other benchmark methods.

### Data

We utilize 8 real-world benchmark data sets from UCI machine learning repository^25^ shown in Table 1 and synthetic data to benchmark our proposed method.

#### Real-world datasets

We have three medium dimension data sets: Breast Cancer Wisconsin-Diagnostic (BCWD), Radar data Goose Bay (Ionosphere), and Breast Cancer Wisconsin-Prognostic (BCWP). Preparation of the data sets for outlier detection acts as other preceding experiments do^12,26^. We remove data with “missing” or “meaningless (especially for attributes having the same value across all data records)” values. Furthermore, we only label the most minority data points as “outlier class”. In *Pendigits*, we test digit “0” to the rest of digits. While keeping the frequencies of the other classes equal, we reduced the number of records of “0”.

If we do not preliminarily process data sets, the group of objects with wrong or duplicate value are large enough to interference the experiment and may makes result not interpretable. After preparation, *BCWD* data sethas 30 attributes; Ionosphere data set has 32 attributes; *BCWP* data set has 33 attributes; And a preprocessed 129-dimensional data set: *arrhythmia*. Note that, the label information of outliers was not used in the discovery process, but used in drawing performance curves. We will choose Euclidean distance as the distance function all throughout our experiments to find the similarity between objects.Data preprocessing affect the result substantially. A brief description of data set used in experiments is shown in Table 2.

**Table 2:** A brief description of data set used in experiments.

Name	#Instances	#Attributes	Benign records	Outlier records	Percentage of outliers
Ann-Thyroid	3772	6	3679	93	2.47%
Arrhythmia	420	129	402	18	4.29%
Breast(Prognostic)	198	33	151	47	23.74%
Breast(Diagnostic)	569	30	357	212	37.26%
Diabetes	768	8	500	268	34.90%
Glass	214	7	205	9	4.20%
Ionosphere	351	32	225	126	35.90%
Pendigits	6870	16	6714	156	2.27%

Relatively large absolute values easily dominate the distance calculation and lead to the loss of information hidden in other important attributes. To counter this effect, the min–max normalization is deployed. The Eq. (5) demonstrate the normalization formula, which is performed for each attribute individually and *n* is the number of attributes.

5 $$X_{norm} = \frac{x\left( i \right) - \min \left( i \right)}{{\max \left( i \right) - \min \left( i \right)}},i = 1, \ldots ,n$$

These data are obtained from UCI Machine Learning Repository^25^. A brief description of data sets illustrated in Table 1. Data sets that used in our experiment are all normalized to interval [0*,*1] by attributes. And the normalization method is described in Eq. (5).

#### Synthetic datasets

Synthetic datasets are suitable for evaluating the performance of scalability since it is convenient to manipulate the deviation, mean and the subspace dimensionality of data. Moreover, generating artificial data has another advantage that we can shape the correlation coefficient between attributes. For experiments on synthetic data. First, we use a collection of benchmarking data sets published by Keller et al.^17^. Then, we generated a data set consist of large number of records (up to 20 K) to evaluate the data size scalability of algorithms in our experiments. Before utilizing ELKI data generator^24^, we modify the data generation scripts from http://www.dbs.ifi.lmu.de/cms/Research/SNN/DataSets with decreasing 640 dimensions to 40 dimensions and generating more records. In dimension scalability experiment, the collection of synthetic datasets are with dimensions range from 10 to 100. Each dataset has 1000 records. Outliers are generated hiding in subspaces with 2–5 dimensions. The outliers were generated in such a way that they are not easily observable in any lower dimensional subspace projection which makes detection more challenging. Synthetic data sets in arff Format^27^ files are available in supplementary material for repetitive of HiCS^17^.

In data size scalability experiment, we will produce a few artificial data sets which have 40 attributes, size from 1000 to 20,000 instances. Each data set has 2% outlier points. Outliers in these data set deviated in 4–5 dimensional subspaces.

### Metric for evaluation

In this section, we describe the metrics of measurement used in the evaluation of different outlier detection algorithms. Previous studies have proposed the confusion matrix^28^ to classify the results of a classification algorithm. In the scenario of outlier detection, outliers will be marked as the positive class, i.e. if an algorithm identified an observation as an outlier correctly, then this is credited with a TP (True Positive). As the proportion of true positive increase, the outcome is better. Otherwise, a wrong classification which incorrectly identified a benign record as an outlier will be considered as an FP (False Positive). When the FP rate decrease, it shows that the algorithm is more accurate. Similarly, FN and TN also have a corresponding definition. FN (False Negative) is the case where the outlier is misclassified to the normal point. TN (True Negative) is defined as the case where the normal point is correctly classified. Table 3 shows the confusion matrix.

**Table 3:** Confusion matrix.

	Ground truth positive	Ground truth negative
Predicted positive	TP	FP
Predicted negative	FN	TN

Nevertheless, it is hard to reflect the effective of a detector only by calculating single pair of sensitivity and specificity. Outlier detection is known as a class imbalance problem since real-world data sets on which mined are common with imbalanced class distributions^29^. ROC (receiver operating characteristic) curve^30^ can be illustrated as follows: Confusion matrix the trade-off between sensitivity and false-positive rate(1-specificity) in imbalanced problem. It is an effective visual means for evaluating and comparing the performance of a predictive function. For example, given a ranking of the objects according to their outlier score, a perfect outlier detection method would first return all outliers followed by all remaining objects. Using the outlier scores as a ranking criterion, ROC curves (receiver operating characteristic) are the means of choice to compare the performance of different methods.

A ROC curve starts from the bottom left corner to the top right and ends at top right corner. The diagonal line from the bottom left to the top right corner indicates a random guess prediction. And an ideal ROC curve is start at (0, 0) then goes along sensitivity-axis to (0, 1) ending at (1, 1). However, it is unrealistic to reach that curve in a real-world outlier detection process.

A common scenario in practical outlier detection applications is that knowing little about the number of outliers. When increasing the number of outliers to retrieve, sensitivity and 1-specificity increase simultaneously. Thus, in order to compare the performance of different algorithm predictive functions, AUC (Area *Under the receiver operating characteristic Curve*) is admitted to tackling this issue. It is useful to depict the sensitivity of methods particularly when the target distribution is imbalanced, as in the outlier detection tasks. Consequently, we use the AUC in following experiments to evaluate our proposed method.

An AUC value of 1.0 means a perfect separation because the area under the ideal ROC curve equals to 1. An AUC value of 0.5 corresponds to random guessing. Since all competitors rely on the parameter *k* specifying the number of the nearest neighbors to be considered, we will compare ROC AUC value under the condition of k with a fixed value (= 100).

In order to measure the statistical uncertainty of the results, we introduced the 95% Confidence Interval (CI) of the AUC value and also performed the bootstrap hypothesistest proposed by Hanley and McNeil^31^ with the experimental result to obtain the corresponding p-value. Python programming language is utilized for mathematical statistics. The *pandas*, *scipy.stats*, *pROC* and *sklearn.metrics* packages were used to complete the statistics of data and results.

### Experiments on real-world data

To validate our approach, we hereby present experimental results on real-world datasets. We compare EPOD with state-of-the-art subspace outlier detection methods: HiCS, OutRank, SOD and OUTRES. Additionally, we also compared EPOD with full-space distance-based(*k*NN), and density-based (LOF) approaches.

#### Entropy-based subspace searching with LOF

In this experiment, comparisons between full space Local Outlier Factor (LOF), HiCS and our proposed method are performed. Authors did not publish source code of HiCS. Therefore, we take the results of HiCS from their paper^17^ shown in Table 3. Moreover, we set parameters of LOF and entropy-based subspace searching as same as they reported in supplementary material for repeatability.

Parameters are set as follows: *k*(*minPts*) = 100. When our method searches interesting subspaces, we use *P* = 25%, *N* = 50%, *ω* = 9.0, and *ϵ* = 0.02. To demonstrate that EPOD is able to select a small number of most interesting subspaces which are good for top *N* outlier detection, and make result comparable, we restrict the number of most interesting subspaces up to 100 of all subspaces. i.e. if the methods in experiment produce 500 subspaces, the proportion(*P* = 25%) of subspaces is more than 100, then the parameter *P* is disabled and only top 100 of these subspaces will be kept for further process.

In this experiment, the result reflects these two subspace methods perform well on these data sets. The *minPts* parameter in the LOF algorithm has properties of upper and lower bounds. However, in our experiments, a fixed k value is chosen for comparison purposes. So, the results reported by HiCS are a little different from the results we reproduce. Nonetheless, HiCS still has a good performance on these datasets. Our proposed method EPOD also gets good results, except on data set Breast(Diag)(89.53%). However, comparing with full space method LOF on Breast(Diag)(86.94%), EPOD still shows better quality. As we can see, the ROC AUC value of EPOD is consistently higher than that of the LOF on each data set.

As the measurement of interestingness in HiCS is based on an implicit notion of density, it may only be appropriate for density-based outlier scores. Hence, in following section, instead of utilizing LOF, we choose *k*NN to discriminate outliers conceal in these datasets. Outlier Detection with LOF: AUC on real-world data is shown in Table 4.

**Table 4:** Outlier detection with LOF: AUC on real-world data.

Data set	Detection method %AUC			
	LOF	HiCS*	HiCS	EPDO
ANN-thyroid	86.16	95.11	91.28	95.11
Arrhythmia	62.92	62.29	52.67	63.44
Breast (prognostic)	56.42	59.31	55.18	59.56
Breast (Diagnostic)	86.94	94.23	76.04	89.53
Diabetes	70.98	72.47	66.87	70.93
Glass	76.86	80.05	76.80	78.92
Ionosphere	77.97	82.34	81.70	84.34
Pendigits	93.54	95.04	94.29	94.47

*Results reported in original paper^17^.

The initial experimental result shows that the proposed validation method, concretely, using the subspaces generated by EPOD to identify the top-n outlier, works effectively in finding the most interesting subspace for outlier detection. While LOF and *k*NN are very competitive in lower dimensional data sets, their performance considerably deteriorates with higher dimensionality while EPOD remains very stable at optimal values.

#### Entropy-based subspace searching with kNN

In this paper, our main focus is to evaluate outlier detection quality EPOD with distance-base outlier scoring function. Here, we use *k*NN distance to score the outlierness of observations. Additionally, subspace methods SOD and OutRank will be added in comparison.

HiCS is an ensemble method for outlier detection in high-dimensional data. It suggests that they^17^ decoupled subspace search and outlier ranking process, and can perform with other outlier scoring function not only LOF but also distance-based scoring function like *k*-Nearest Neighbors(*k*NN) algorithm. Parameters in HiCS are set: *candidate cutoff* = 400, Monte Carlo iteration limit *m* = 50 and *Alpha* = 0*.*1 (Parameter settings are suggested by^17^).

Since HiCS integrated with a random sampling process, we perform experiment on each dataset 10 times, then pick up the average value of the results. SOD in this experiment is with parameters: SOD.*SharedNearestNeighbor* = 100; SOD.*kNN* = 100. For a fair comparison, *k* in *k*NN algorithm are set to the same value. And we choose fixed *k* = 100 of *k*NN algorithm; Entropy threshold for subspace selection *ω* = 8.5, interest gain threshold *ϵ* = 0.1, Most interesting subspace proportion *P* = 25, and Top percentage object in voting *N* = 50% in this experiment. These parameter sets yield consistently good results in following section IV-D so we recommend choosing it accordingly. A comparison of effective performance of different outlier detection method on 8 real-world data sets using *k*-Nearest Neighbor distance: ROC AUC value is shown in Table 5.

**Table 5:** A comparison of effective performance of different outlier detection method on 8 real-world data sets using *k*-nearest neighbor distance: ROC AUC value.

Data set	Detection method % AUC [95% CI high, low]|p-value					
	*k*NN	HiCS	Enclus	SOD	OutRank	EPOD
ANN-thyroid	94.42 [91.12,96.49]	95.22 [89.91,98.91]	90.96 [89.90,92.61]	92.24 [89.29,94.53]	80.22 [79.49,83.07]	98.62 [95.64,99.80]
	< 0.001	< 0.001	< 0.001	< 0.001	< 0.001	< 0.001
Arrhythmia	64.2 [60.90,67.70]	62.3 [52.00,74.86]	65.8 [62.85,68.46]	72.4 [58.11,85.23]	54.5 [51.64,56.90]	67.91 [64.92,69.58]
	0.059	0.081	0.045	0.024		0.031
Breast (prognostic)	50.1 [48.55,54.58]	55.7 [49.32,59.51]	54.7 [52.34,56.74]	51.7 [47.29,56.93]	51.41 [48.61,53.85]	57.91 [56.89,58.46]
	0.830	0.171	0.078	0.951	0.5312	0.057
Breast (diagnostic)	87.47 [82.10,90.40]	92.17 [90.65,95.99]	92.80 [90.75,94.78]	84.40 [78.47,89.45]	67.58 [64.46,72.34]	89.84 [85.46,93.25]
	< 0.001	< 0.001	< 0.001	< 0.001	0.058	< 0.001
Diabetes	73.16 [72.04,74.85]	72.90 [70.80,74.98]	71.15 [70.64,72.63]	64.88 [61.90,66.51]	51.12 [48.74,53.75]	71.60 [69.72,72.52]
	0.002	0.003	0.002	0.052	0.931	0.002
Glass	78.80 [76.55,80.58]	75.82 [71.30,79.25]	79.73 [77.60,80.02]	76.75 [72.52,81.72]	74.61 [72.78,76.64]	82.33 [79.90,84.51]
	< 0.001	< 0.001	< 0.001	< 0.001	< 0.001	< 0.001
Ionosphere	82.87 [81.09,83.39]	76.54 [71.25,82.88]	82.37 [80.95,85.38]	80.22 [78.46,82.25]	82.93 [80.63,84.60]	84.22 [82.48,86.90]
	< 0.001	0.004	< 0.001	< 0.001	< 0.001	< 0.001
Pendigits	95.50 [93.93,96.60]	94.38 [91.79,96.58]	94.29 [92.92,95.58]	83.95 [80.89,87.46]	78.98 [72.02,80.95]	94.20 [90.92,95.08]
	< 0.001	< 0.001	< 0.001	< 0.001	< 0.001	< 0.001

The results of these real-world experiments are listed in Table 5. On *Pendigits* data all algorithms receive high scores. We confirm that, for a well-chosen value of k, *k*NN performs best. The best *k* on data set Diabetes is 86 (*AUC* = 73*.*22%). In our designate *k* equals 100 is much closer than on other datasets. Similarly, results on data set *Pendigits*also demonstrated this phenomenon. In addition, the best *k* value can be found in VIII. It can be concluded from Table 5 that our proposed method outperforms overall. It preforms as the best algorithm on four data sets (*Thyroid* 98.62%; *Breast* 57.91%; *Glass* 82.33%; *Ionosphere* 84.22%). Compared with other approaches in rest four experiments, the divergence of AUC value is less than 4%. In general, the proposed method shows a high average quality of AUC, which implies that it is favorable.

### Sensitivity of parameters

In this section, we will discuss the impact of parameters selecting in EPOD algorithm.

#### Effect of the parameters to EPOD

We conduct the experiments on a single data set to study how the entropy threshold *ω* and the interest threshold *ϵ* affect the performance of EPOD with the Top 25% subspaces ranking. The parameters *ω* and *ϵ* help us to select subspace among the subspaces with dense clusters lying inside and subspaces with significant correlation between pair-wise attributes respectively. We choose to perform experiment on Arrhythmia Database from the UCI Machine Learning Repository. This data set contains 420 instances of samples from two classes. The first class is “Normal” contains 402 records, and the other marked as “outlier” with 18 instances. The procedure of these experiments settings follows the steps described in previous section. Note that in this section, subspace pruning and ranking are not applied.

**Figure 2:**
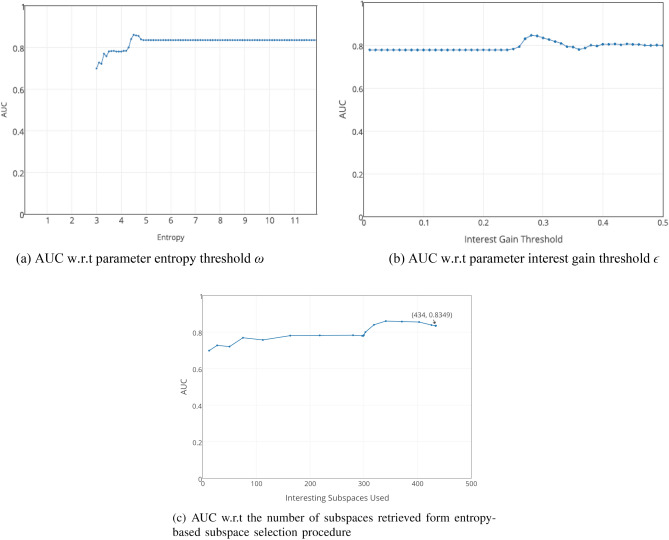
AUC w.r.t parameters *ω* and *ϵ* on data set Arrhythmia.

The experiment results are shown in Fig. 2. From Fig. 2a, we can see that for the data set the AUC of EPOD is monotonically increasing when *ω* ∈ [3,5], and then it reached a stable state when *ω* ∈ [5,12]. And from Fig. 2b, we can see that the AUC of EPOD increase with the increase of subspaces produced by selection process. Note that when number of interesting subspaces reached at 400, the AUC decreased slightly. Afterwards, AUC values stayed at 0.84 with number of interesting subspaces 434. It should be noted that, due to the effect of algorithm pruning, in the case of increasing the parameters of *ω*, and will not produce more subspace. As a matter of fact, for an outlier detection task we prefer higher AUC and more stable results. Hence, in a practical application, one should select the value of *ϵ* as large as possible in acceptable computational cost to guarantee effectiveness. In our experiments, EPOD works well when *ω* ∈ [6,12]. So, combining Fig. 2a and b, our experiments suggest that the value of *ω* is suitable when it is within [6,12].

#### Effect of the ranking method to EPOD

In order to further investigate the parameter *ω*, *ϵ* with regard to interesting subspace ranking method jointly impact on outlier detection result, we conduct experiment on the same data set in previous subsection, with *ω* ∈ [6,12] incremental stepping 0.1; *ϵ* ∈ [0.01,0.5] with incremental stepping 0.01.

**Figure 3:**
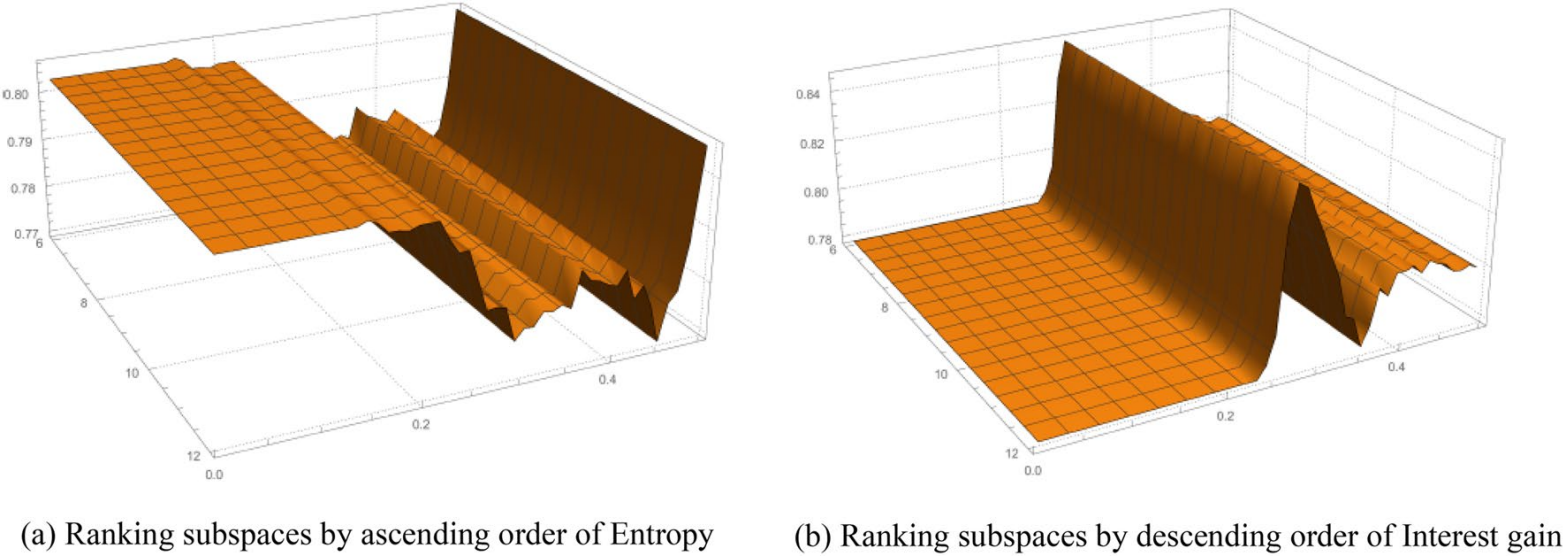
AUC w.r.t subspace ranking methods.

The results are depicted in Fig. 3. We utilize entropy and interest gains of subspaces as criteria for ranking the selected subspaces. As these two metrics have been employed to identify interesting subspaces before, we can efficiently obtain their values after the subspace mining procedures without any additional calculations.

From Fig. 3a, we can see that, with the increase of *ϵ*, the AUC value of EPOD is relatively stable before *ϵ* = 0*.*22. From Fig. 3b, we can see that the AUC value of EPOD are generally stable except when *ϵ* is around0.3.

According to the experiments in Fig. 2, results indicate that our algorithm is not affected by *ω* and *ϵ* considerably. The outcome AUC from Fig. 3 manifests its stability, and we only need to investigate the results on other datasets when *ω* = {7*,*8*,*9}. Further, we can conclude that proposed algorithm EPOD does not need a host of subspaces in subsequent steps. Thus, by changing these two parameters, the number of subspaces returned from entropy-based subspace selection can also varies slightly on real-world datasets. Overall, changes of number of subspaces do not bring significant impact on the overall accuracy of the algorithm.

#### Effect of the parameters on real-world datasets

In this experiment, we choose a range of parameters which are suggested in previous results. And set them in entropy-based subspaces selection process to investigate the impact on quality of outcomes on eight UCI real-world datasets. For entropy threshold *ω*, as suggested, we set in the range of [7,9] with interval 0.1. For interest gain threshold *ϵ*, which is less than 0.2, values are set to {0.1, 0.01, 0.02}.

Afterwards, in order to confirm this hypothesis, an experiment on real-world datasets with restricted parameters is conducted. The results of the experiment are shown in Table 6.

**Table 6:** Impact on entropy-based subspace parameters selection.

ROC AUC (%) on real-world datasets									
Entropy *ω*	7			8			9		
Interest gain *ϵ*	0.001	0.01	0.02	0.001	0.01	0.02	0.001	0.01	0.02
Ann-thyroid	96.27	95.62	95.11	96.27	95.62	95.11	96.27	95.62	95.11
Arrhythmia	64.40	64.40	64.40	64.40	64.40	64.40	64.40	64.40	64.40
Breast (prognostic)	51.82	51.82	51.82	51.82	51.82	51.82	51.82	51.82	51.82
Breast (diagnostic)	88.67	88.67	88.67	88.67	88.67	88.67	88.67	88.67	88.67
Diabetes	70.92	70.92	70.92	70.92	70.92	70.92	70.92	70.92	70.92
Glass	78.92	78.92	78.92	78.92	78.92	78.92	78.92	78.92	78.92
Ionosphere	85.54	85.54	85.54	85.54	85.54	85.54	85.54	85.54	85.54
Pendigits	93.24	93.24	93.30	93.24	93.24	93.30	93.24	93.24	93.30

### Scalability experiments

**Figure 4:**
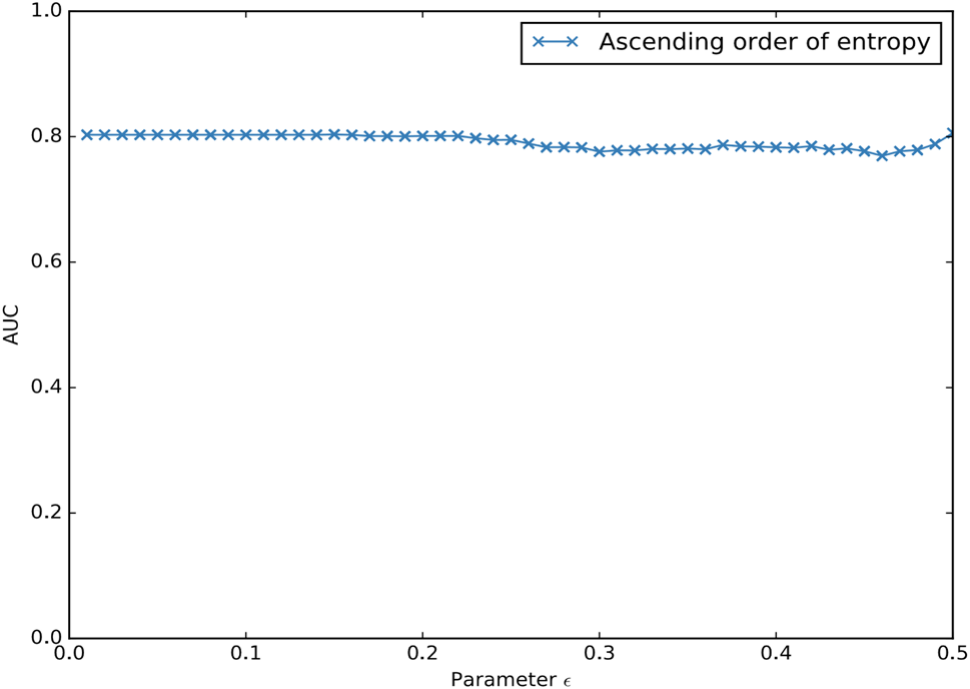
Ranking subspaces by ascending order of entropy.

**Figure 5:**
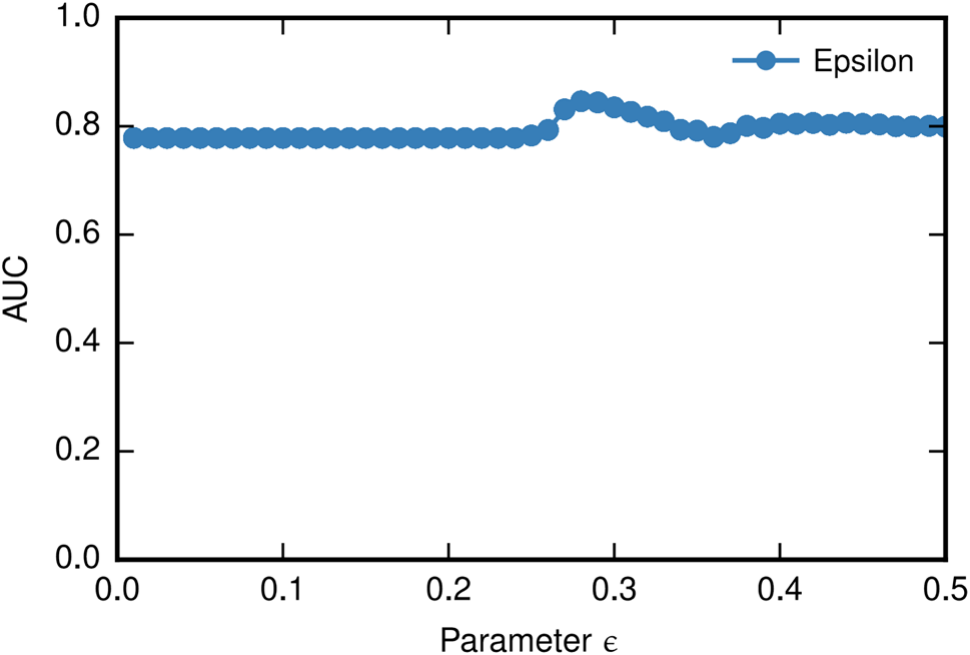
Ranking subspaces by descending order of interest gain.

To evaluate the runtime of EPOD, experiments are performed on synthetic data with high dimensionality. By integrating our proposed algorithm in ELKI, we perform scalability toward increasing dimension, which is ranged from 10 to 100. Ranking subspaces by ascending order of entropy is shown in Fig. 4, and Ranking subspaces by descending order of interest gain is shown in Fig. 5.

**Figure 6:**
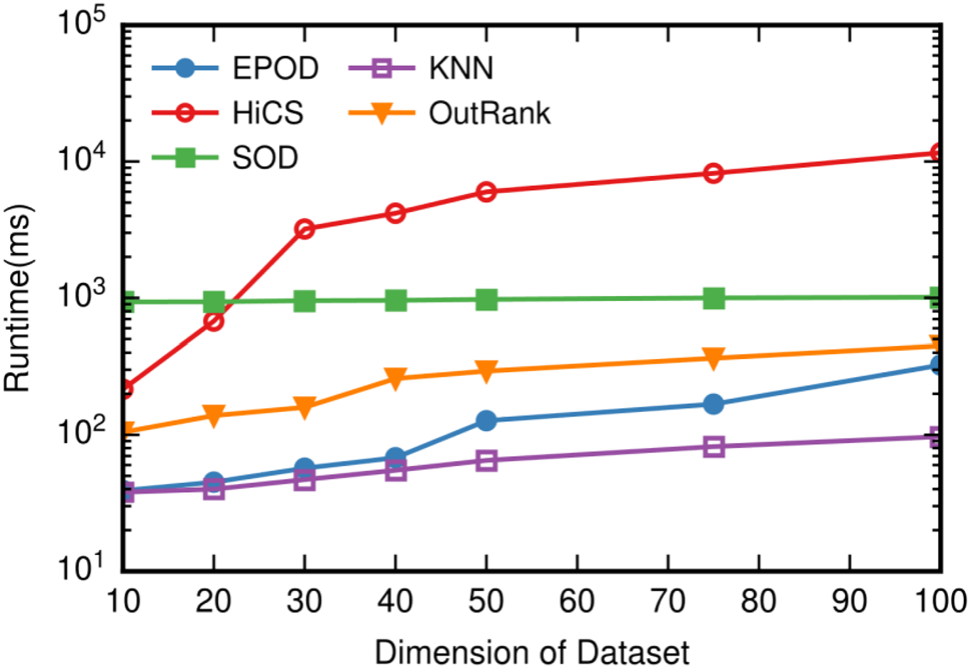
Runtime w.r.t. dimensionality.

And all parameters are set as the same in the previous experiment. The experiment is repeated 10 times, and the average of runtime is recorded. We depict the runtime of each algorithm with regard to increasing dimensionality in Fig. 6.

Significant differences were observed between EPOD and HiCS in Fig. 6. Both two algorithm have the exponential incremental performance overhead. However, the runtime of HiCS increased drastically as the dimension are larger than 20. It should be noted that we did not implement HiCS ourselves, alternatively, we utilized ELKI^24^ framework to conduct experiments using HiCS method. EPOD typically performs better than HiCS. Besides, the direct application of *k*NN or LOF methods to high dimensional problems often results in unexpected performance and qualitative costs due to the curse of dimensionality.

## Discussion

### Outlier detection approaches

Density-based outlier detection methods are founded on the assumption that the density surrounding a normal data point is comparable to that of its neighbors. Commonly used techniques include Local Outlier Detection^32–34^, which has given rise to numerous variations^23,33,35,36^.

**Figure 7:**
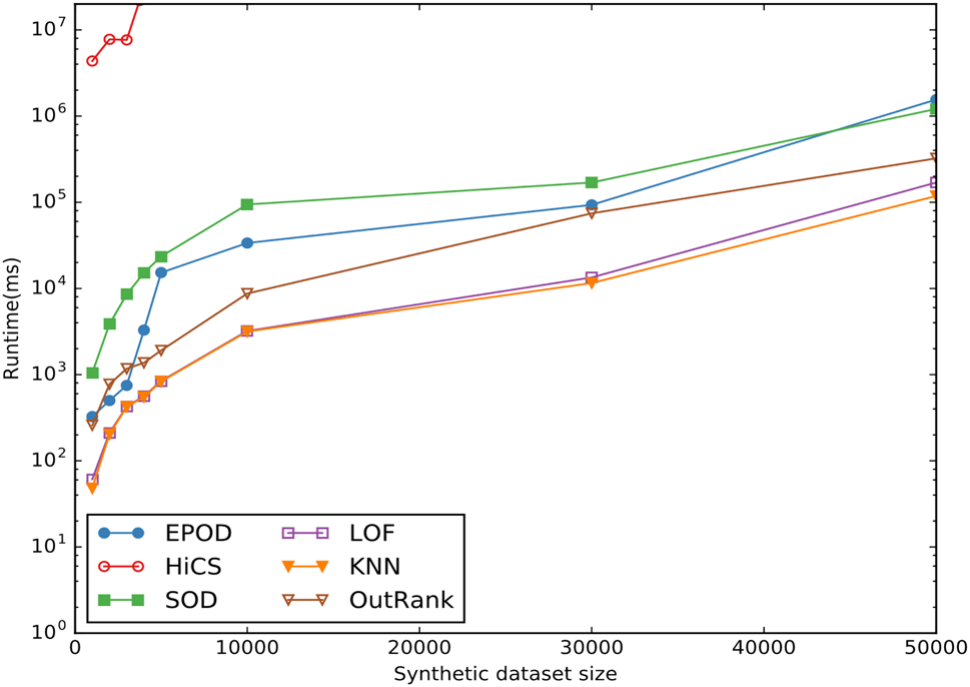
Runtime w.r.t. dataset size.

As shown in Fig. 7, compared to other approaches, density-based methods are more locally sensitive and tend to achieve higher accuracy. A notable representative is Local Outlier Factor (LOF)^32^, which computes a relative density score using an extended k-nearest neighbor approach. LOF effectively indicates the unusualness of an instance and serves as an outlier index. This density-based approach has demonstrated strong performance across various applications and has influenced subsequent works in the literature.

On the other hand, distance-based approaches, assume that normal data examples emerge from dense neighborhoods, while outliers correspond to isolated points. These methods assign an outlier score to each instance using a robust variant of the Mahalanobis distance, measuring the distance between each instance and the main body of the data distribution. Instances located far from the rest of the data are identified as outliers. Other methods falling under this category include Knorr’s unified approach^22^. A limitation of distance-based methods is their susceptibility to the curse of dimensionality problem. The number of parameters in these models grows quadratically with the number of dimensions, rendering them less suitable for high-dimensional data. In such cases, high-dimensional approaches are proposed to address the sparsity of data samples and the lack of meaningful neighborhoods. Typical methods in this class either adopt an invariant distance measurement, such as angle-based outlier detection^37^, or project the data into a lower-dimensional subspace, as seen in grid-based subspace outlier detection^12^ and Sparse PCA.

### Subspace outlier detection approaches

Generalized dimensionality reduction techniques, such as Principal Components Analysis (PCA), are commonly used in classification to achieve lower-dimensional subspace projections. However, these techniques are not specifically designed as preprocessing steps for outlier discovery. In other words, outliers in these algorithms are often considered as byproducts or even disturbances when performing selection on lower-dimensional manifolds. Consequently, the lack of sensitivity to outliers is due to the focus on obtaining robust eigenvectors, making these approaches unsuitable for direct outlier detection in high-dimensional data.

One of the earliest approaches to address high-dimensional subspace outlier detection was presented by Aggarwal and Yu^12,13^. This approach bears resemblance to a grid-based subspace clustering approach but differs in that it searches for sparse grid cells instead of dense ones. The method identifies objects contained within these sparse grid cells as outliers through an evolutionary search process. Aggarwal and Yu assume that data points are uniformly distributed in projected subspaces and that outliers are associated with data cubes in projected subspaces that are significantly sparser than expected. However, a major issue with their approach arises when dealing with increasing dimensionality, as the expected value of a grid cell quickly becomes too low to identify significantly sparse grid cells effectively.

Furthermore, a critical drawback of their approach lies in the unrealistic assumption that the distribution of data points is uniform. In reality, when data points are not uniformly distributed, sparse data cubes can be as normal as dense data cubes, leading to unreliable results. Additionally, the approach does not take into account relationships between attributes. The correlation among attributes can significantly impact the density of data cubes in the projected spaces, further compromising the interpretability of the results. Nevertheless, this pioneering work represents the first attempt to tackle the problem from a novel perspective.

Enclus^20^ is a subspace clustering method based on the CLIQUE algorithm. Unlike directly measuring coverage and density, Enclus calculates these characteristics using entropy. The algorithm leverages the understanding that a subspace with denser clusters exhibits lower entropy. The cluster ability of a subspace is defined using three criteria: coverage, density, and correlation, all of which can be measured using entropy. As cell density increases, entropy decreases. Additionally, under certain conditions, entropy decreases with increasing coverage, and correlation is measured using an interest metric. Interest is defined as the difference between the sum of entropy measurements for a set of dimensions and the entropy of the multi-dimensional distribution. Higher interest values indicate stronger correlation between dimensions, while an interest value of zero indicates independent dimensions.

Similar to Apriori^38^, Enclus employs a bottom-up approach, as used in CLIQUE, to mine significant subspaces. Pruning is performed by exploiting the downward closure property of entropy (below a threshold ω) and the upward closure property of interest to identify minimally correlated subspaces. If a subspace is highly correlated (above threshold ϵ), all of its superspaces must also be correlated. Since non-minimally correlated subspaces might still be of interest, Enclus searches for interesting subspaces by calculating interest gain and identifying subspaces with entropy exceeding ω and interest gain surpassing ϵ. Once interesting subspaces are identified, clusters can be determined using the same methodology as CLIQUE or any other existing clustering algorithm. Enclus also shares much of the flexibility seen in CLIQUE.

Another algorithm, called High-dimension Outlying Subspace Detection (HighDOD), was proposed in^39^ to identify outlying subspaces. It determines the outlier degree based on the sum of distances between a point and its k nearest neighbors. The data set is randomly sampled, and the search begins at data points with high outlier degrees.

OutRank can analyze the results of various subspace clustering algorithms, including grid-based and density-based methods. By focusing on the stability of clusters across different subspaces, OutRank aims to mitigate statistical bias and identify outliers with greater accuracy. The outlierness is determined by the frequency of an object being recognized as part of a cluster and is also related to the correlation among the subspaces in which the object resides. However, OutRank assumes strong redundancy in clustering and treats outliers merely as a side-product of the clustering algorithm, potentially leading to a large set of outliers.

SOD (Subspace Outlier Detection) identifies outliers in subspaces without explicitly referencing a clustering result. Instead, a reference set is used to possibly define a subspace cluster (or a part of such a cluster) implicitly. If a query point significantly deviates from the subspace of the reference set, it is considered a subspace outlier with respect to the corresponding subspace. The subspace distance outlier score is not based on a binary decision of outlier vs. inlier but rather a normalized score. However, some limitations of SOD include challenges in finding a good reference set and the oversimplification of score normalization.

In high contrast subspaces (HiCS), which refers to subspaces with high contrast based on the correlation among their attributes. HiCS aggregates the LOF scores for a single object across all "high contrast" subspaces, suggesting the use of alternative outlier measures instead of LOF. In these subspaces, outliers are non-trivial and deviate from the correlation trend exhibited by the majority of data in the subspace. However, combining LOF scores from subspaces with varying dimensionality without score normalization introduces a Bias Scores problem. Moreover, the naive combination of scores could benefit from ensemble reasoning. Identifying interesting subspaces may yield different outcomes depending on the outlier ranking measures used, and HiCS’ measure of interest relies on an implicit notion of density, making it more suitable for density-based outlier scores. Despite this decoupling, HiCS offers valuable insights into the issue of subspace selection, which is the main focus of their study.

## Conclusion

In this paper, we have explored the application of entropy-based clustering with notable sensitivity in mining clusters characterized by both denseness and small size. We presented an intuitive definition for outlier detection in high-dimensional space. Thenwe introduced EPOD, an efficient algorithm for outlier detection, which utilizes entropy as the criterion for subspace selection. Through experimental comparisons with existing methods, EPOD has exhibited superior performance.

The proposed algorithm exhibits remarkable effectiveness on medium-dimensional datasets while showcasing excellent scalability on high-dimensional datasets. By employing entropy-based selection, EPOD ranks numerous meaningful subspaces. Subsequently, we leveraged the standardized k-nearest neighbor (kNN) distance to calculate the outlierness of observations across different subspaces. Although EPOD's performance with LOF does not match that of kNN, considering the trade-off between efficiency and scalability, it remains a promising approach.

Drawing on the statistical implications of the outlier score, we propose the algorithm to vote for the outlierness points in significant and interesting subspaces. To the best of our knowledge, while a few previous works have utilized entropy as a metric to mine clusters embedded in subspace or full space, no prior method has harnessed it for the specific problem of outlier detection in subspace. This highlights the novelty and potential significance of our approach in the field of outlier detection.

## Data Availability

The data that support the findings of this study are available from the corresponding author upon reasonable request.

## References

[1] Fawcett T, Provost F (1997). Adaptive fraud detection. Data Min. Knowl. Discov..

[2] Mazel J, Casas P, Fontugne R, Fukuda K, Owezarski P (2015). Hunting attacks in the dark: clustering and correlation analysis for unsupervised anomaly detection. Int. J. Netw. Manag..

[3] Podgorelec, V., Hericko, M. and Rozman, I. Improving mining of medical data by outliers prediction. In *18th IEEE Symposium on Computer-Based Medical Systems, 2005. Proceedings*, pp. 91–96 (2005).

[4] Hawkins, D. M. “Introduction,” in *Identification of Outliers*, ser. Monographs on Applied Probability and Statistics (Springer Netherlands, 1980), pp. 1–12. 10.1007/978-94-015-3994-41

[5] Barnett V, Lewis T (1994). Outliers in Statistical Data.

[6] Hodge VJ, Austin J (2004). A survey of outlier detection methodologies. ArtifIntell. Rev..

[7] Chandola V, Banerjee A, Kumar V (2009). Anomaly detection: A survey. ACM Comput. Surv..

[8] Zimek A, Schubert E, Kriegel H-P (2012). A survey on unsupervised outlier detection in high-dimensional numerical data. Stat. Analy. Data Min..

[9] Aggarwal CC, Aggarwal CC (2013). High-dimensional outlier detection: The subspace method. Outlier Analysis.

[10] Beyer K, Goldstein J, Ramakrishnan R, Shaft U, Beeri C, Buneman P (1999). “When is “nearest neighbor” meaningful?. Database Theory—ICDT’99, ser. Lecture Notes in Computer Science.

[11] Garey MR, Johnson DS (1979). Computers and Intractability: A Guide to the Theory of NP-Completeness.

[12] Aggarwal, C. C. and Yu, P. S. Outlier detection for high dimensional data. In *Proceedings of the 2001 ACM SIGMOD International Conference on Management of Data*, ser. SIGMOD ’01. (ACM, 2001), 37–46. 10.1145/375663.375668

[13] Aggarwal C, Yu S (2005). An effective and efficient algorithm for high-dimensional outlier detection. VLDB J..

[14] Zimek A, Campello RJ, Sander J (2014). Ensembles for unsupervised outlier detection: Challenges and research questions a position paper. SIGKDD Explor. Newsl..

[15] Kriegel H-P, Kroger P, Schubert E, Zimek A, Theeramunkong T, Kijsirikul B, Cercone N, Ho T-B (2009). Outlier detection in axis-parallel subspaces of high dimensional data. Advances in Knowledge Discovery and Data Mining.

[16] Muller, E., Assent, I., Iglesias, P., Mulle, Y. and Bohm, K.Outlier Ranking via subspace analysis in multiple views of the data. In *Proceedings of the 2012 IEEE 12th International Conference on Data Mining* (IEEE Computer Society, 2012) 529–538. 10.1109/ICDM.2012.112

[17] Keller, F., Muller, E. and Bohm, K. HiCS: High contrast subspaces for density-based outlier ranking. In *2012 IEEE 28th International Conference on Data Engineering (ICDE)*, 1037–1048 (2012).

[18] Muller, E., Assent, I., Steinhausen, U. and Seidl, T. OutRank: Ranking outliers in high dimensional data. In *IEEE 24th International Conference on Data Engineering Workshop, 2008. ICDEW 2008*, 600–603 (2008).

[19] Muller, E., Schiffer, M. and Seidl, T. Adaptive Outlierness for Subspace Outlier Ranking. In *Proceedings of the 19th ACM International Conference on Information and Knowledge Management*, ser. CIKM ’10 (ACM, 2010) 1629–1632, 00012. 10.1145/1871437.1871690

[20] Cheng, C.-H., Fu, A. W. and Zhang, Y. Entropy-based Subspace clustering for mining numerical data. In *Proceedings of the Fifth ACM SIGKDD International Conference on Knowledge Discovery and Data Mining*, ser. KDD ’99 (ACM, 1999) 84–93. 10.1145/312129.312199

[21] Ramaswamy, S., Rastogi, R. and Shim, K. Efficient algorithms for mining outliers from large data sets. In *Proceedings of the 2000 ACM SIGMOD International Conference on Management of Data*, ser. SIGMOD ’00 (ACM, 2000), 438, 01243. 10.1145/342009.335437

[22] Knorr, E. M. and Ng, R. T. Algorithms for mining distance-based outliers in large datasets. In *Proceedings of the 24rd International Conference on Very Large Data Bases*, ser. VLDB ’98 (Morgan Kaufmann Publishers Inc., 1998), 392–403. http://dl.acm.org/citation.cfm?id=645924.671334

[23] Zhang K, Hutter M, Jin H, Theeramunkong T, Kijsirikul B, Cercone N, Ho T-B (2009). A New local distance-based outlier detection approach for scattered real-world data. Advances in Knowledge Discovery and Data Mining.

[24] Achtert, E., Kriegel, H.-P., Schubert, E. and Zimek, A. Interactive data mining with 3d-parallel-coordinate-trees. In *Proceedings of the 2013 ACM SIGMOD International Conference on Management of Data*, ser. SIGMOD ’13 (ACM, 2013), 1009–1012. 10.1145/2463676.2463696

[25] Lichman, M. *UCI Machine Learning Repository*. University of California, Irvine, School of Information and Computer Sciences. http://archive.ics.uci.edu/ml (2013).

[26] Muller, E., Schiffer, M. and Seidl, T. Statistical selection of relevant subspace projections for outlier ranking. In *2011 IEEE 27th International Conference on Data Engineering (ICDE)*, pp. 434–445, (2011).

[27] Hall M, Frank E, Holmes G, Pfahringer B, Reutemann P, Witten IH (2009). The WEKA data mining software: An update. SIGKDD Explor. Newsl..

[28] Foody, G. M. Status of land cover classification accuracy assessment (2001).

[29] Tan P-N, Steinbach M, Kumar V (2005). Introduction to Data Mining.

[30] Bradley AP (1997). The use of the area under the ROC curve in the evaluation of machine learning algorithms. Pattern Recogn..

[31] Hanley JA, McNeil BJ (1982). The meaning and use of the area under a receiver operating characteristic (ROC) curve. Radiology.

[32] Breunig MM, Kriegel H-P, Ng RT, Sander J (2000). LOF: Identifying density-based local outliers. ACM SIGMOD Rec..

[33] Papadimitriou, S., Kitagawa, H., Gibbons, P. and Faloutsos, C. LOCI: Fast outlier detection using the local correlation integral. In *19th International Conference on Data Engineering, 2003. Proceedings*, pp. 315–326, (2003).

[34] Tang, J., Chen, Z., Fu, A. W.-C. and Cheung, D. W.-L. Enhancing effectiveness of outlier detections for low density patterns. In *Proceedings of the 6th Pacific-Asia Conference on Advances in Knowledge Discovery and Data Mining*, ser. PAKDD ’02 (Springer-Verlag, 2002), 535–548. http://dl.acm.org/citation.cfm?id=646420.693665

[35] Jin W, Tung AKH, Han J, Wang W, Ng W-K, Kitsuregawa M, Li J, Chang K (2006). Ranking outliers using symmetric neighborhood relationship. Advances in Knowledge Discovery and Data Mining.

[36] Kriegel, H.-P., Kroger, P., Schubert, E. and Zimek, A. LoOP: Local outlier probabilities. In *Proceedings of the 18th ACM Conference on Information and Knowledge Management*, ser. CIKM ’09 (ACM, 2009), 1649–1652, 10.1145/1645953.1646195

[37] Kriegel, H.-P., Hubert, M. S and Zimek, A. Angle-based outlier detection in high-dimensional data. In *Proceedings of the 14th ACM SIGKDD International Conference on Knowledge Discovery and Data Mining*, ser. KDD ’08 (ACM, 2008), pp. 444–452. 10.1145/1401890.1401946

[38] Agrawal, R. and Srikant, R. Fast Algorithms for mining association rules in large databases. In *Proceedings of the 20th International Conference on Very Large Data Bases*, ser. VLDB ’94, pp. 487–499 (Morgan Kaufmann Publishers Inc., 1994). http://dl.acm.org/citation.cfm?id=645920.672836

[39] Zhang J, Wang H (2006). Detecting outlying subspaces for high-dimensional data: The new task, algorithms, and performance. Knowl. Inf. Syst..

